# Altered Fractional Amplitude of Low-Frequency Fluctuation in Major Depressive Disorder and Bipolar Disorder

**DOI:** 10.3389/fpsyt.2021.739210

**Published:** 2021-10-13

**Authors:** Yan Qiu, Min Yang, Sujuan Li, Ziwei Teng, Kun Jin, Chujun Wu, Xuelei Xu, Jindong Chen, Hui Tang, Jing Huang, Hui Xiang, Wenbin Guo, Bolun Wang, Haishan Wu

**Affiliations:** ^1^Department of Psychiatry, China National Technology Institute on Mental Disorders, National Clinical Research Center for Mental Disorders, The Second Xiangya Hospital of Central South University, Changsha, China; ^2^Department of Radiology, The Second Xiangya Hospital of Central South University, Changsha, China

**Keywords:** bipolar disorder (BD), major depressive disorder (MDD), fractional amplitude of low-frequency fluctuation (fALFF), cognitive function, resting-state functional magnetic resonance imaging (rs-fMRI)

## Abstract

**Background:** Discriminating between major depressive disorder (MDD) and bipolar disorder (BD) remains challenging and cognitive deficits in MDD and BD are generally recognized. In this study, the fractional amplitude of low-frequency fluctuation (fALFF) approach was performed to explore neural activity and cognition in first-episode, drug-naïve BD and MDD patients, as well as the relationship between altered fALFF values and clinical or psychometric variables.

**Methods:** A total of 21 BD patients, 25 MDD patients, and 41 healthy controls (HCs) completed clinical assessments and resting-state functional magnetic resonance imaging (rs-fMRI) scans in this study. The rs-fMRI data were analyzed by fALFF method and Pearson correlation analyses were performed between altered fALFF values and clinical variables or cognition. Support vector machine (SVM) was adopted to identify the three groups from each other with abnormal fALFF values in the brain regions obtained by group comparisons.

**Results:** (1) The fALFF values were significantly different in the frontal lobe, temporal lobe, and left precuneus among three groups. In comparison to HCs, BD showed increased fALFF values in the right inferior temporal gyrus (ITG) and decreased fALFF values in the right middle temporal gyrus, while MDD showed decreased fALFF values in the right cerebellar lobule IV/V. In comparison to MDD, BD showed decreased fALFF values in bilateral posterior cingulate gyrus and the right cerebellar lobule VIII/IX. (2) In the BD group, a negative correlation was found between increased fALFF values in the right ITG and years of education, and a positive correlation was found between decreased fALFF values in the right cerebellar lobule VIII/IX and visuospatial abilities. (3) The fALFF values in the right cerebellar lobule VIII/IX may have the ability to discriminate BD patients from MDD patients, with sensitivity, specificity, and accuracy all over 0.70.

**Conclusions:** Abnormal brain activities were observed in BD and MDD and were related with cognition in BD patients. The abnormality in the cerebellum can be potentially used to identify BD from MDD patients.

## Introduction

Major depressive disorder (MDD) and bipolar disorder (BD) are both common and highly prevalent mood disorders with social disabilities and heavy financial burdens (1, 2). Epidemiological studies showed that half of diagnosed MDD patients could be actually unrecognized BD patients (3). Moreover, BD patients usually seek medical help for depressive symptoms, and the phenomenology of depressive state in BD is similar to the symptoms in MDD (4). Thus, discriminating between BD and MDD is challenging. This challenge leads to misdiagnoses and long-delayed diagnoses in BD (5). In recent years, exploring pathophysiological differences between BD and MDD is urgently needed (6), and increasing studies were conducted to find biomarkers to identify BD from MDD (7). However, the results remained inconsistent, and the goal of distinguishing BD from MDD has not been achieved.

Cognitive dysfunction in BD and MDD has been widely recognized (8, 9). Cognitive deficits mostly in psychomotor speed, visual learning and memory, attention, and executive function were reported in MDD patients (10). Cognitive deficits mainly in the domains of attention, memory, and executive function were identified in BD patients (11). One study found that BD and MDD displayed similar deficits in immediate memory, language, attention, and delayed memory (12). In another study, BD patients showed more severe deficits in all cognitive domains than MDD (13). However, Poletti et al. found that BD inpatients were only worse in verbal fluency than MDD inpatients (14). At all, the results of comparison in cognition between BD and MDD are inconsistent, and few studies compared cognitive function between BD and MDD in the early stage.

Resting-state functional magnetic resonance imaging (rs-fMRI) is a good tool to explore brain function. The rs-fMRI data could be used for functional connectivity (FC) analysis, regional homogeneity (ReHo) analysis, and measuring the amplitude of low-frequency fluctuation (ALFF) value. The FC analysis approach is difficult to determine which is the abnormal brain region, when abnormal FC exists between two brain areas (15). The ReHo analysis approach can find the abnormal activities in the nearest neighboring voxels, but cannot measure the functional connections in the whole brain regions. Sensitive to physiological noise, the ALFF approach was improved to the fractional amplitude of low-frequency fluctuation (fALFF) approach (16). Furthermore, the fALFF method can be more sensitive and specific to reflect regional spontaneous brain activity based on blood oxygen level-dependent (BOLD) signals (17). Recently, more and more studies applied the fALFF approach in BD (18) and MDD (19, 20) patients. One MRI study reported that BD showed increased gray matter volume (GMV) in the right-sided dorsolateral prefrontal cortex than MDD and proposed that different mechanisms mediate pathophysiological processes of BD and MDD (21). However, another MRI study reported that compared to healthy controls (HCs), both BD and MDD patients displayed decreased GMV as well as decreased resting-state FC in the frontal-limbic system. The results of this study suggested that there were more similarities than differences in functional and structural alterations between drug-naïve BD and MDD patients (22). To date, few studies have directly compared BD and MDD using the fALFF method and there is heterogeneity in the results. More studies are needed to distinguish BD from MDD, especially in the early stages of the diseases.

Among those rs-MRI studies of BD and MDD (23, 24), the relationship between clinical variables and the parameters in rs-MRI was usually explored. In these studies, the clinical variables mostly referred to scale scores, duration of illness, onset age of illness, and number of episodes, and the rs-MRI parameters usually involved FC values, ReHo values, ALFF values, and fALFF values. To our knowledge, few studies have combined cognitive function with rs-MRI parameters such as fALFF values in BD and MDD. Investigating the relationship between cognition and fALFF values could deepen the understanding of mood disorders and further provide information for diagnosis and treatment.

As a computational algorithm, the support vector machine (SVM) has been popular in classifying patients (25). It learns and recognizes the main features of labeled training data and predicts the labels of testing targets. The SVM method can detect patterns that are difficult to discern from large or complex datasets (26); thus, it is suitable for identifying BD patients and MDD patients.

In this study, first-episode and drug-naive BD or MDD patients as well as HCs were recruited. The fALFF values were calculated to compare neural activity among the three groups. According to the abovementioned studies, we hypothesized that the three groups may have significant fALFF differences in the temporal lobe and cerebellum when compared with each other, and these brain regions with altered fALFF values may serve as markers for identifying BD and MDD patients. Furthermore, we conducted correlation analyses to see whether the altered fALFF values were associated with clinical variables.

## Methods and Materials

### Subjects

Forty-six right-handed patients, aged 18–55 years, were continuously recruited from the Second Xiangya Hospital of Central South University, Changsha, Hunan province, China. Twenty-one of them were BD patients and 25 of them were MDD patients. All the patients met the diagnostic criteria of Diagnostic and Statistical Manual of Mental Disorders, Fifth Edition (DSM-5) by using the Structured Clinical Interview, patient version. All the patients had never received medication, physical therapy, or psychotherapy, and the illness course was <5 years. The exclusion criteria for MDD included the following: (1) patients with organic mental diseases or any history of craniocerebral trauma; (2) other psychiatric diseases except for MDD and BD; (3) alcohol or substance abuse; (4) severe physical diseases such as cardiovascular, kidney, or liver diseases; (5) pregnancy; (6) age younger than 18 years old or older than 55 years old; and (7) the 17-item Hamilton Rating Scale for Depression (HRSD) scores ≤ 17. The exclusion criteria for BD were the Young Mania Rating Scale (YMRS) scores ≥7 and all those exclusion criteria for MDD. The anxiety symptoms and cognition of the patients were assessed with the Hamilton Anxiety Scale-14 (HAMA-14), Stroop task, and the Repeatable Battery for the Assessment of Neuropsychological Status (RBANS).

Forty-one right-handed HCs were recruited from the local community through advertisements. The HCs were well-matched with the patients for age, sex ratio, and years of education. They were screened through the Structured Clinical Interview for DSM-5 (SCID), non-patient edition, in order to exclude the presence of psychiatric disorders. The exclusion criteria for HCs included the following: (1) any first-degree relative has a current or past psychiatric illness and (2) the same abovementioned exclusion criteria for patients. Cognitive function of HCs was assessed through the Stroop task and RBANS.

### Data Acquisition

The magnetic resonance imaging (MRI) was performed on a Philips 3-T scanner. Participants were required to lie still on the foam padding and keep awake without thinking anything. Headphones were used to reduce the interference of noise during the scanning process. Functional images were acquired with a gradient-echo echo-planar imaging (EPI) sequence. The following sequence parameters were applied: repetition time/echo time (TR/TE) = 2,000/30 ms, slices = 30, data matrix = 64 × 64, flip angle (FA) = 90°, field of view (FOV) = 240 mm, slice thickness/gap = 4/0.4 mm, and 250 volumes lasting for 500 s.

### Data Preprocessing

The rs-fMRI data preprocessing was conducted in MATLAB software using the Data Processing Assistant for Resting State fMRI (DPARSF) (27), basic edition. Slice timing was corrected for acquisition delay between slices. All the participants should have a head motion with maximum displacement <2 mm in any direction of *x, y*, and *z*, as well as rotation <2° in any angular dimension. Then, the images were normalized to the standard Montreal Neurological Institute (MNI) echo-planar imaging space, and resampled to a voxel size of 3 × 3 × 3 mm^3^. The resulting data were temporally band-pass filtered (0.01–0.08 Hz), linear detrended, and spatially smoothed with a Gaussian kernel of 4-mm full-width at half-maximum (FWHM). For each subject, Friston-24 head motion parameters and signals from white matter and cerebrospinal fluid were removed to eliminate their influence on fALFF. Framewise displacement (FD) of head position was calculated (28) and the time points with FD > 0.2 mm were removed to eliminate the residual effects.

### fALFF Analysis

The fALFF analysis was performed by DPARSF software (27). Fast Fourier transform (FFT) was used to transform the time course of each voxel into the frequency domain without bandpass filtering and then the power spectrum was obtained. The square root was calculated at each frequency of the power spectrum, and the average square root was acquired in the 0.01–0.08 Hz range for each voxel. The fALFF value was the ratio of the power spectrum in the low-frequency range to the power spectrum in the entire frequency range, so the sum of the amplitudes over 0.01–0.08 Hz range was divided by that over entire frequency range. Finally, for the purpose of standardization, the fALFF maps was obtained through the fALFF of each voxel divided by the global mean fALFF value (17).

### Classification Analysis

SVM classifier works in the form of supervised learning and can work well with small training sample size (29). The discrimination margin is gotten by the way of iterations. During each iteration, samples with class labels are used to calculate the classifier except for one sample for testing. After multiple iterations, the decision function is set up. These processes are called the leave-one-out (LOO) cross-validation strategy, which is adopted for optimality assurance. The kernel type applied in this study was the default Gaussian kernel. The brain regions with altered fALFF values were obtained by group comparisons, and then the abnormal fALFF values were extracted from these regions for further classification analysis. The LIBSVM software^1^ was used to identify BD patients or MDD patients from HCs and identify BD patients from MDD patients. The results of sensitivity, specificity, and accuracy for classification were obtained. The sensitivity refers to the proportion of patients correctly classified, the specificity refers to the controls correctly classified, and the accuracy refers to the proportion of all the samples correctly classified (30).

### Statistical Analysis

Chi-square tests were used to assess differences in categorical variables among groups. One-way analyses of variance (ANOVAs) were used to assess difference in continuous variables among three groups and *post-hoc t*-tests were performed between each two groups. The significant level among three groups was set at *p* < 0.05 (two-tailed). The magnitude of the differences in continuous variables was explored by calculating the effect size Cohen's *d*. The values of Cohen's *d* over 0.8 are defined as high effect sizes, while medium and small effect sizes are 0.5–0.8 and 0.2–0.5, respectively (12). The significant level between each of the two groups was set at *p* < 0.05 (two-tailed), Bonferroni corrected.

Using age and years of education as covariates, the whole-brain fALFF values were compared among three groups by one-way ANOVA *via* voxel-wise cross-subject statistics in DPARSF software. The significance threshold was set at *p* < 0.05 for multiple comparisons using Gaussian Random Field (GRF) theory. *Post-hoc t*-tests were performed to compare the whole-brain fALFF values between each of the two groups with age and years of education as covariates. The significance level was set at *p* < 0.05 for *post-hoc* multiple comparisons, corrected by Bonferroni. Pearson correlation analysis was performed between the brain regions with abnormal fALFF values and clinical or psychometrics variables in patients.

## Results

### Demographics and Clinical Characteristics

Twenty-one BD patients, 25 MDD patients, and 41 HCs were included in this study. All the patients were at a depressive episode during the study. One BD patient, two MDD patients, and one healthy control were excluded in the final analysis for excessive head movements during rs-fMRI scans. No difference in age and gender distribution (Table 1) was found among three groups. BD patients displayed higher education and HAMD-17 scores than MDD, with medium effect size in education (Tables 1, 2).

**Table 1:** Characteristics of the subjects.

	**BD (*n* = 20)**	**MDD (*n* = 23)**	**HCs (*n* = 40)**	**χ^**2**^/*F***	***p***
Sex (male/female)	5/15	10/13	15/25	1.64	0.440
Age (years)	22.80 ± 4.30	22.78 ± 4.40	21.20 ± 1.80	2.32	0.105
Education (years)	15.25 ± 1.07	14.26 ± 1.79	14.65 ± 0.86	3.49	**0.035**
FD	0.08 ± 0.01	0.08 ± 0.01	0.08 ± 0.01	0.40	0.675
HAMD-17	23.26 ± 5.10	26.17 ± 5.04	–	3.53	0.067
HAMA-14	25.94 ± 7.55	19.74 ± 5.93	–	9.09	**0.004**
YRMS	3.94 ± 2.33	–	–	–	–
Immediate memory	42.83 ± 8.15	35.30 ± 9.17	50.28 ± 6.56	27.85	**<0.001**
Visuospatial	31.72 ± 4.74	33.17 ± 4.59	35.08 ± 3.36	4.82	**0.011**
Language	29.00 ± 4.82	27.30 ± 5.00	30.92 ± 4.38	4.52	**0.014**
Attention	70.39 ± 7.93	63.35 ± 8.50	75.46 ± 9.28	14.00	**<0.001**
Delayed memory	48.61 ± 4.65	46.78 ± 6.90	54.21 ± 4.60	16.32	**<0.001**
RBANS total	224.39 ± 19.56	205.91 ± 26.36	246.08 ± 20.76	24.70	**<0.001**
Stroop word	98.83 ± 14.56	87.87 ± 20.11	104.69 ± 13.49	8.26	**0.001**
Stroop color	70.89 ± 13.32	61.48 ± 18.06	77.59 ± 13.66	8.53	**<0.001**
Stroop color-word	43.22 ± 8.15	38.30 ± 9.06	45.23 ± 11.10	3.58	**0.032**
Stroop total score	212.94 ± 30.89	187.65 ± 35.28	227.51 ± 31.24	11.11	**<0.001**

*Chi-square tests were performed in categorical variables among three groups. One-way analyses of variance (ANOVAs) were performed in continuous variables among three groups*.

*Bold typeface indicates statistical significance*.

*BD, bipolar disorder; MDD, major depressive disorder; FD, framewise displacement; HAMD-17, Hamilton Depression Scale-17; HAMA-14, Hamilton anxiety Scale-14; YRMS, Young Mania Rating Scale; RBANS, Repeatable Battery for the Assessment of Neuropsychological Status*.

**Table 2:** *Post-hoc* pairwise comparisons of clinical variables and cognition.

	**BD vs. HCs**		**MDD vs. HCs**		**BD vs. MDD**	
	**(*****n*** **= 20)**		**(*****n*** **= 23)**		**(*****n*** **= 40)**	
	**Cohen's *d***	***p***	**Cohen's *d***	***P***	**Cohen's *d***	***p***
Education (years)	0.64	0.237	0.31	0.692	0.66	**0.031**
Immediate memory	1.05	**0.002**	1.97	**<0.001**	0.86	**0.006**
Visuospatial	0.87	**0.011**	0.49	0.235	0.31	0.744
Language	0.42	0.409	0.78	**0.012**	0.35	0.714
Attention	0.57	0.113	1.35	**<0.001**	0.85	**0.031**
Delayed memory	1.21	**<0.001**	1.34	**<0.001**	0.31	0.799
RBANS total	1.07	**0.002**	1.75	**<0.001**	0.79	**0.024**
Stroop word	0.42	0.540	1.04	**<0.001**	0.62	0.078
Stroop color	0.50	0.315	1.05	**<0.001**	0.59	0.127
Stroop color-word	0.20	1.000	0.67	**0.028**	0.57	0.327
Stroop total score	0.47	0.311	1.22	**<0.001**	0.76	**0.037**

*Post-hoc t-tests were performed between two groups; The significance level was set at p < 0.05, Bonferroni corrected. The p values <0.05 marked with bold style and represent statistical differences in pairwise comparisons*.

*BD, bipolar disorder; MDD, major depressive disorder; HCs, healthy controls; RBANS, Repeatable Battery for the Assessment of Neuropsychological Status*.

All RBANS subtests, Stroop subtests, and BRANS total and Stroop total scores revealed significant differences among three groups (Table 1). In comparison to HCs, BD displayed significantly lower scores in immediate memory, visuospatial abilities, delayed memory, and BRANS total, while MDD displayed lower scores in immediate memory, language, attention, delayed memory, RBANS total, Stroop word, Stroop color, Stroop color-word, and Stroop total. Compared to MDD, BD showed higher scores in immediate memory, attention, Stroop total, and RBANS total (Table 2).

In the comparison between BD and HCs, high effect sizes were found in immediate memory, visuospatial abilities, delayed memory, and BRANS total scores. In the comparison between MDD and HCs, high effect sizes were found in immediate memory, attention, delayed memory, RBANS total, Stroop word, Stroop color, and Stroop total scores, and medium effect sizes were found in language and Stroop color-word. In the comparison between BD and MDD, high effect sizes were found in immediate memory and attention, and medium effect sizes were found in years of education, RBANS total scores, and Stroop total scores (Table 2).

### Differences in fALFF Values Among Groups

**Figure 1:**
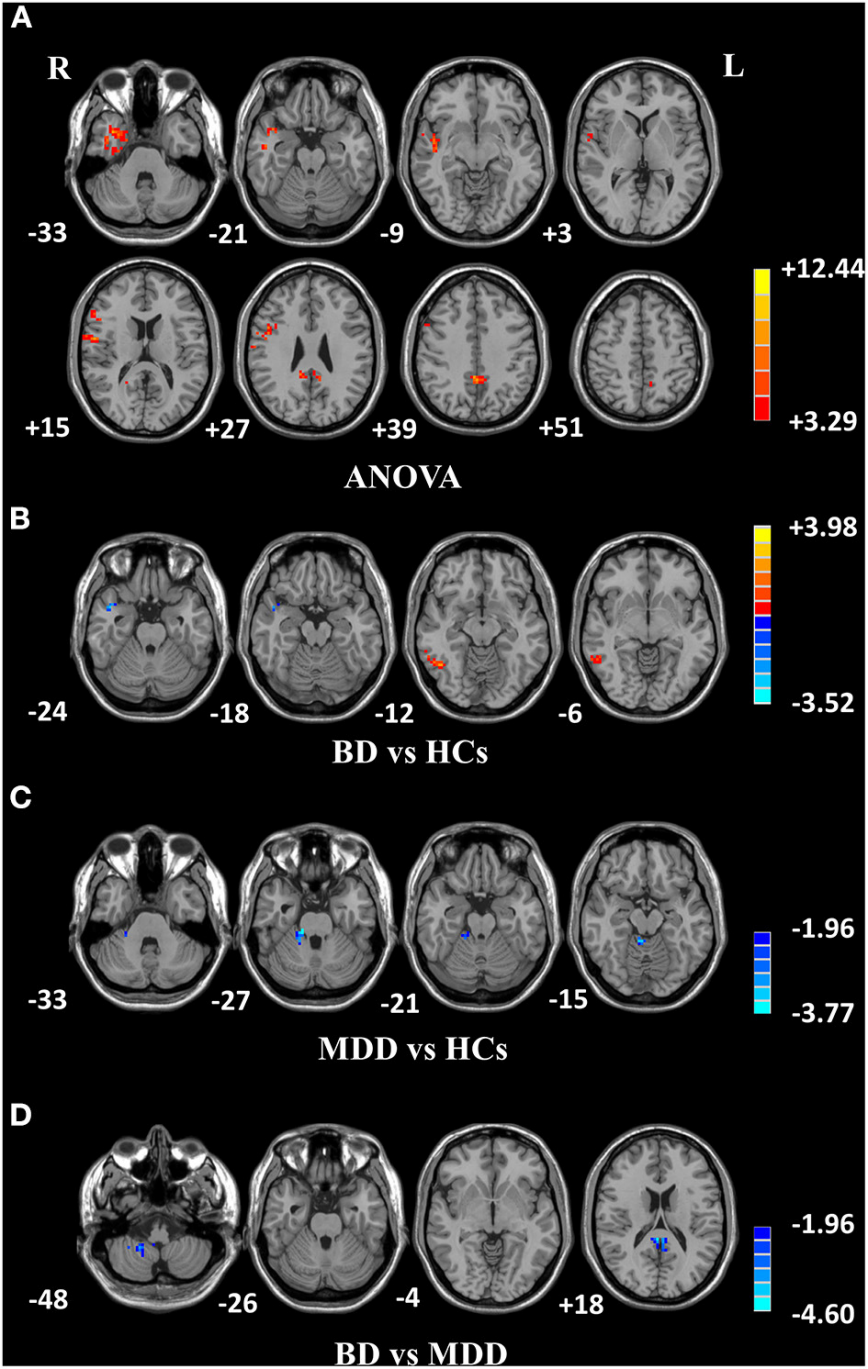
Differences in fALFF values among groups. **(A)** Different fALFF values in the right temporal lobe, right frontal lobe, and left precuneus were observed among three groups. **(B)** Different fALFF values in the right ITG and right MTG were observed between BD and HCs. **(C)** Different fALFF values in the right cerebellar lobule IV/V were observed between MDD and HCs. **(D)** Different fALFF values in bilateral PCG and the right cerebellar lobule VIII/IX were observed between BD and MDD. The color bar represents the *t* values of analyses. Red and blue denote higher and lower fALFF values, respectively. fALFF, fractional amplitude of low-frequency fluctuation; BD, bipolar disorder; MDD, major depression disorder; HCs, healthy controls; L, left hemisphere; R, right hemisphere; ITG, inferior temporal gyrus; MTG, middle temporal gyrus; PCG, posterior cingulate gyrus.

Among the three groups, significantly different fALFF values were detected in the right temporal pole: middle temporal gyrus (MTG); right superior temporal gyrus; right inferior frontal gyrus, triangular part; and left precuneus (PCUN) (Table 3 and Figure 1A). Compared to HCs, BD showed increased fALFF values in the right inferior temporal gyrus (ITG) and decreased fALFF values in the right MTG (Table 3 and Figure 1B), while MDD showed decreased fALFF in the right cerebellar lobule IV/V (Table 3 and Figure 1C). Compared to MDD, BD showed decreased fALFF values in bilateral posterior cingulate gyrus (PCG) and the right cerebellar lobule VIII/IX (Table 3 and Figure 1D).

**Table 3:** Regions with fALFF differences in the BD group, MDD group, and HCs.

**Cluster location**	**Peak (MNI)**			**Number of voxels**	***T*-value**
	***x***	***y***	***z***		
**Difference among three group**					
TPOmid.R/PHG.R/TPOsup.R	36	6	−33	117	8.23
STG.R/MTG.R/FFG.R	45	−6	−12	101	9.61
IFGtriang.R/PreCG.R/PoCG.R	60	24	18	156	11.71
PCUN.L/PCG.R	3	−45	39	148	8.58
**BD>HCs**					
ITG.R	48	−57	−12	70	3.12
**BD < HCs**					
MTG.R	45	6	−24	56	−3.07
**MDD < HCs**					
Cere_ IV/V.R	15	−33	−27	57	−3.77
**BD < MDD**					
Cere_ VIII/IX.R	21	−48	−48	53	−3.69
PCG.R/PCG.L	3	−39	18	94	−4.60

*The significance level was set at p < 0.05 for multiple comparisons corrected by Gaussian Random Field (GRF) theory (voxel significance: p < 0.001, cluster significance: p < 0.05). The significance level for post-hoc comparisons was set at p < 0.05, Bonferroni corrected*.

*BD, bipolar disorder; MDD, major depression disorder; HCs, healthy controls; fALFF, fractional amplitude of low-frequency fluctuation; MNI, Montreal Neurological Institute; TPOmid.R, temporal pole: right middle temporal gyrus; PHG.R, right parahippocampal gyrus; TPOsup.R, temporal pole: right superior temporal gyrus; STG.R, right superior temporal gyrus; MTG.R, right middle temporal gyrus; FFG.R, right fusiform gyrus; IFGtriang.R, right inferior frontal gyrus, triangular part; PreCG.R, right precentral gyrus; PoCG.R, right postcentral gyrus; PCUN.L, left precuneus; PCG.R, right posterior cingulate gyrus; ITG.R, right inferior temporal gyrus; Cere_ IV/V.R, right cerebellar lobule IV/V; Cere_ VIII/IX.R, right cerebellar lobule VIII/IX; PCG.L, left posterior cingulate gyrus*.

### Correlations Between Abnormal fALFF Values and Clinical or Psychometric Variables

**Figure 2:**
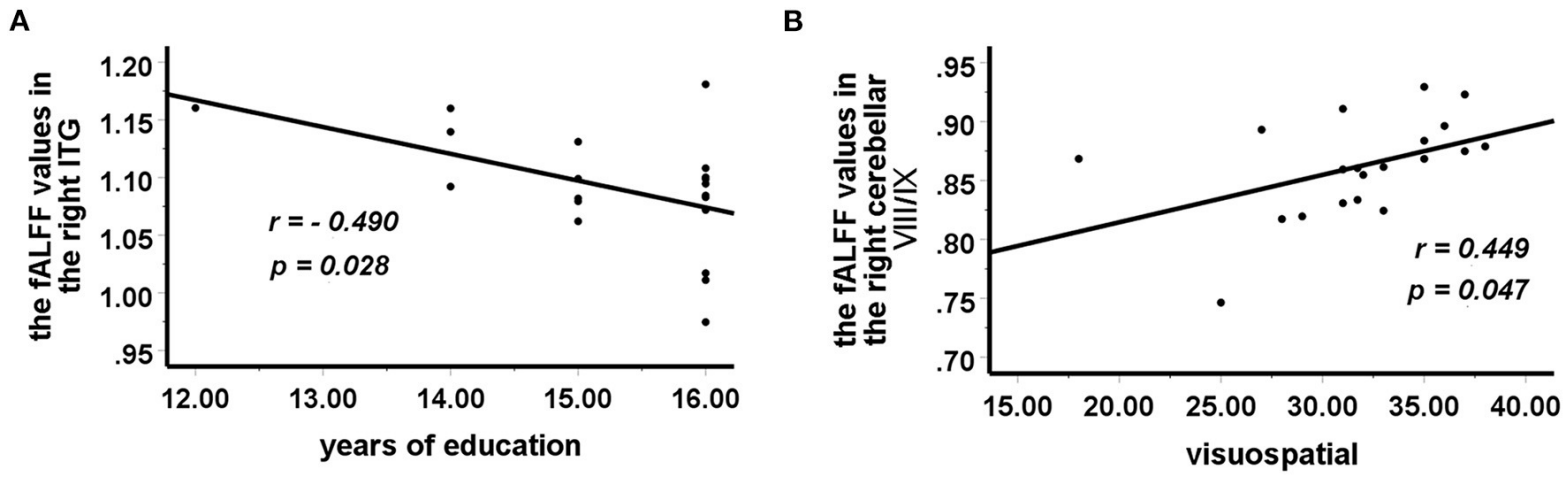
Correlations between abnormal fALFF values and clinical variables. In patients with BD, **(A)** a negative correlation was observed between the fALFF values in the right ITG and years of education, and **(B)** a positive correlation was observed between the fALFF values in the right cerebellar lobule VIII/IX and visuospatial abilities. fALFF, fractional amplitude of low-frequency fluctuation; BD, bipolar disorder; ITG, inferior temporal gyrus.

In the BD group, increased fALFF values in the right ITG were negatively correlated with years of education (*r* = −0.490, *p* = 0.028) (Figure 2A), and decreased fALFF values in the right cerebellar lobule VIII/IX were positively correlated with visuospatial abilities (*r* = 0.449, *p* = 0.047) (Figure 2B). No correlation was observed between altered fALFF values in the brain regions of patients and other clinical variables (age, HAMD scores, HAMA scores, YMRS scores, all RBANS subtests, and RBANS total scores).

### Classification Result

**Figure 3:**
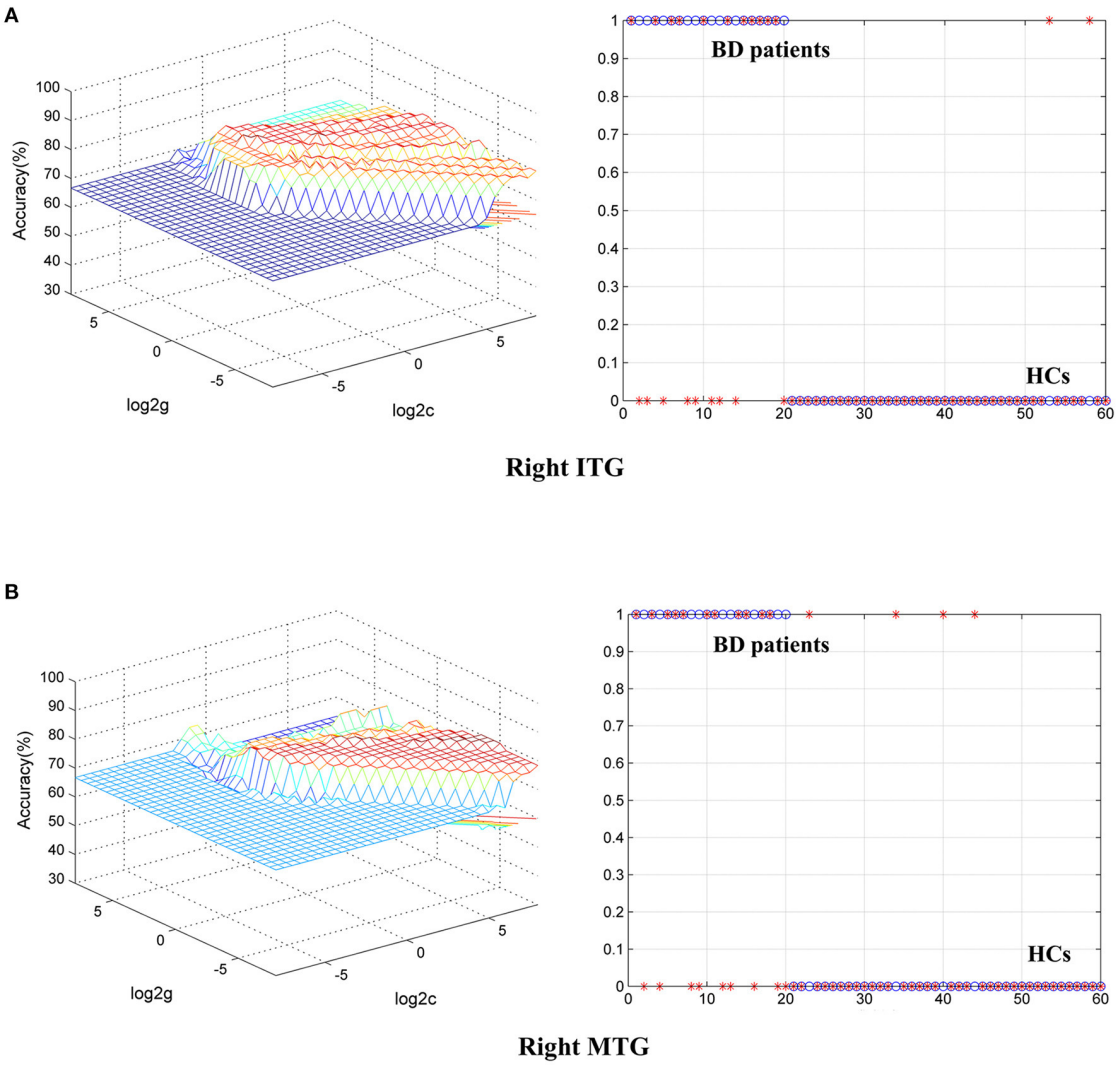
Differentiating BD patients from HCs with SVM. The abnormal fALFF values in right ITG and right MTG were used to perform classification analyses by SVM. **(A,B)** Two graphs composite the results of one region. In each combination, the left graph is a 3D view of accuracy result and the right graph is the classified map. BD, bipolar disorder; HCs, healthy controls; fALFF, fractional amplitude of low-frequency fluctuation; SVM, support vector machine; ITG, inferior temporal gyrus; MTG, middle temporal gyrus; PCG, posterior cingulate gyrus.

**Figure 4:**
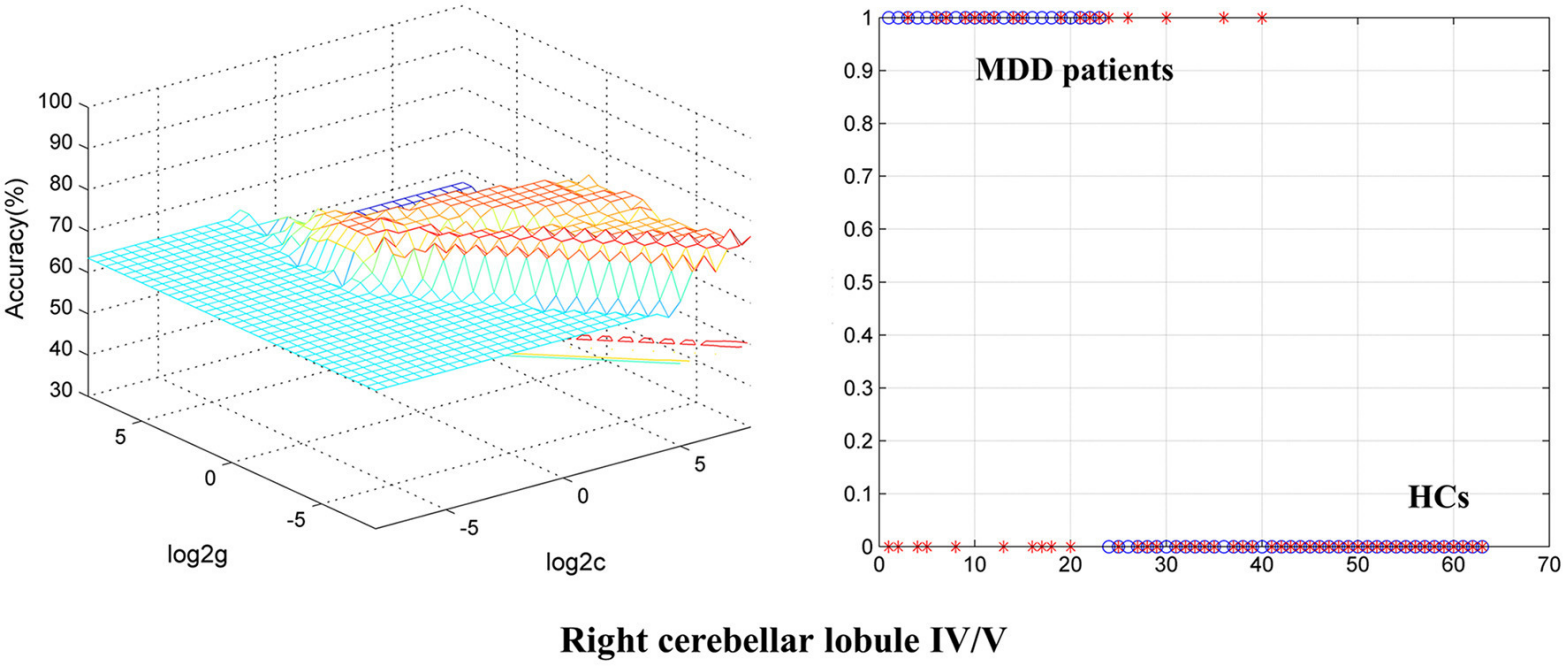
Differentiating MDD patients from HCs with SVM. The abnormal fALFF values in right cerebellar lobule IV/V were used to perform classification analysis by SVM. The left graph is a 3D view of accuracy result and the right graph is the classified map. MDD, major depression disorder; HCs, healthy controls; fALFF, fractional amplitude of low-frequency fluctuation; SVM, support vector machine.

**Figure 5:**
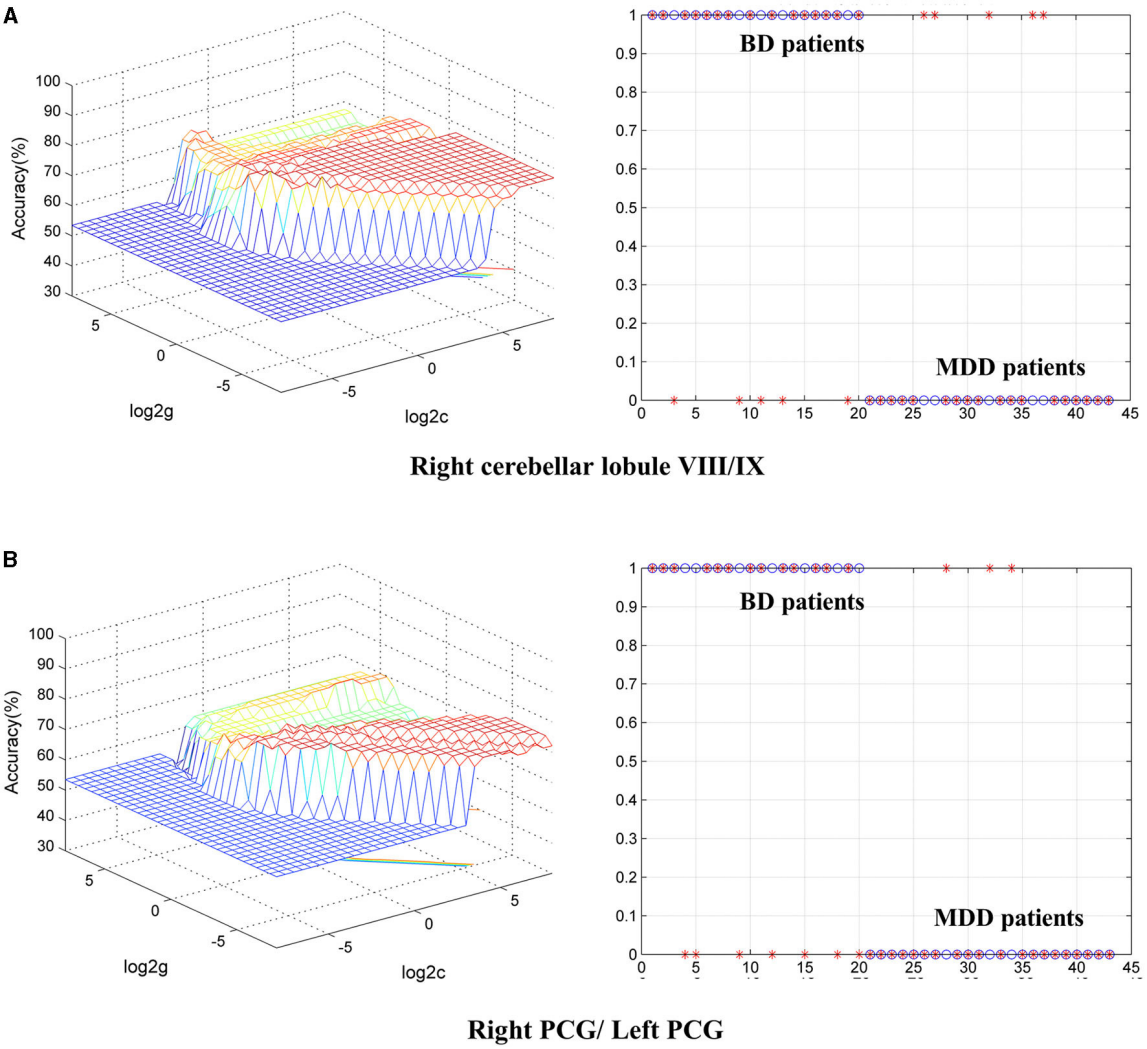
Differentiating BD patients from MDD with SVM. The abnormal fALFF values in bilateral PCG and the right cerebellar lobule VIII/IX were used to perform classification analyses by SVM. **(A,B)** Two graphs composite the results of one region. In each combination, the left graph is a 3D view of accuracy result and the right graph is the classified map. BD, bipolar disorder; MDD, major depression disorder; fALFF, fractional amplitude of low-frequency fluctuation; SVM, support vector machine; PCG, posterior cingulate gyrus.

To identify BD from HCs, the accuracy and specificity of the right ITG were higher than the right MTG, and the sensitivities of the two regions were the same (Table 4 and Figure 3). To identify MDD from HCs, the specificity and accuracy of the right cerebellar lobule IV/V were more than 75.00%, and the sensitivity of it was <60.00% (Table 4 and Figure 4). To identify BD from MDD, higher sensitivity and lower specificity were found in the right cerebellar lobule VIII/IX than bilateral PCG, and the accuracies of the two regions were the same (Table 4 and Figure 5).

**Table 4:** Identifying BD and MDD by using the fALFF values in several regions with SVM method.

**Brian region**	**Classification**	**Sensitivity**	**Specificity**	**Accuracy**
ITG.R	BD, HCs	55.00% (11/20)	95.00% (38/40)	81.67% (49/60)
MTG.R	BD, HCs	55.00% (11/20)	90.00% (36/40)	78.33% (47/60)
Cere_ IV/V.R	MDD, HCs	56.52% (13/23)	87.50% (35/40)	76.19% (48/63)
Cere_ VIII/IX.R	BD, MDD	75.00% (15/20)	78.26% (18/23)	76.74% (33/43)
PCG.R/ PCG.L	BD, MDD	65.00% (13/20)	86.96% (20/23)	76.74% (33/43)

*BD, bipolar disorder; MDD, major depressive disorder; HCs, healthy controls; SVM, support vector machines; fALFF, fractional amplitude of low-frequency fluctuation; ITG.R, right inferior temporal gyrus; MTG.R, right middle temporal gyrus; Cere_ IV/V.R, right cerebellar lobule IV/V; Cere_ VIII/IX.R, right cerebellar lobule VIII/IX; PCG.R, right posterior cingulate gyrus; PCG.L, left posterior cingulate gyrus*.

## Discussion

The aims of this study were to compare cognition and neural activity, as well as explore the relationship between brain activity and clinical characteristics in BD and MDD. As a result, MDD patients displayed more severe and extensive cognitive deficits than BD patients. BD, MDD, and HCs showed statistical differences of fALFF values in the right temporal lobe, right frontal lobe, and left PCUN. Compared with HCs, BD showed increased fALFF values in the right ITG and decreased fALFF values in the right MTG, while MDD showed decreased fALFF values in the right cerebellar lobule IV/V. When compared to MDD, BD showed decreased fALFF values in bilateral PCG and the right cerebellar lobule VIII/IX. Moreover, increased fALFF values in the right ITG were negatively correlated with years of education in BD. The decreased fALFF values in the right cerebellar lobule VIII/IX were positively correlated with visuospatial abilities in BD, and this brain region can be potentially used to identify BD from MDD by SVM analysis. In all, our study added an expanding literature to the hypothesis of different neural mechanisms mediating BD and MDD and preliminarily provided information for the pathologies of BD and MDD.

Stroop scores are golden measurements for executive function including inhibition and attention (31). In this study, all subtests of RBANS and Stroop task, RBANS total, and Stroop total scores were significantly different among three groups. The results of pairwise comparisons revealed that MDD patients showed more severe and extensive cognitive deficits than BD patients. These indicated that cognition at depressive state may be different between BD and MDD, including memory, visuospatial abilities, attention, and executive function. A meta-analysis reported that bipolar depression performed severe deficits in simple reaction time, Trail Making Test A (TMT-A) processing speed, and word list learning than unipolar depression (32). In later life, BD patients showed lower scores in motor speed, verbal fluency, attention and processing speed, and lower composite scores than MDD (33). These studies above as well as ours supported the view that different cognitive functions exist between BD and MDD. However, another study reported that BD and MDD patients showed similar deficits in psychomotor speed, working memory, visual memory, attention switching, and verbal fluency (34). The contradictory conclusions in comparing cognition between BD and MDD patients need further exploration in the future.

Compared with HCs, BD patients showed a dissociation pattern of spontaneous neural activities in the temporal lobe, with decreased activity in the right MTG and increased activity in the right ITG. The MTG is the gyrus between the superior temporal sulcus and the inferior temporal sulcus. As one key region of the semantic system (35), it is associated with semantic memory (36), creativity (37), color discrimination (38), action observation, and word processing (39). The decreased activity found in the right MTG revealed that there may be functional inhibition or injury in this region and could thus lead to cognitive impairment such as verbal memory. One study reported that the cortical thickness of the MTG was associated with verbal hallucinations and was negatively correlated with hallucination severity in schizophrenia (40). Unfortunately, we did not assess the presence of hallucination in BD patients. The ITG, located below the MTG, is found to work with several cortical regions and is considered to participate in vision, language, and emotion (41, 42). Nowadays, neuroinflammation is considered to be an adaptive immune process (43) and can partly explain the increased neural activities in BD (44). It is possible that the decreased activity in the right MTG causes compensatory immune activation in the right ITG and then the altered neural activities influence cognition. The alteration of brain activity in the cerebellum in this study suggests that both injury and adaptive functional alterations may exist in the temporal lobe of BD.

Compared with HCs, MDD displayed decreased fALFF values in the right cerebellar lobule IV/V. It suggests that MDD patients showed decreased neural activity in the cerebellum. The cerebellum has fibrous connections with many brain regions like prefrontal cortex and limbic system (45), and it is involved in emotion and cognition other than motor regulation (46, 47). One study reported that MDD showed increased fALFF values in the left Crus I and left cerebellar lobule VI relative to HCs (48). This study also found decreased FC between the left Crus I and right hippocampus, but increased FC between the left Crus I and left inferior parietal lobule and between the left cerebellar lobule VI and bilateral ITG. In another study, MDD patients showed decreased ReHo in the left cerebellum posterior lobe, right FFG, left PHG, and right PoCG compared to HCs (45). These results as well as ours supported the view that there may be functional abnormality in the cerebellum of MDD patients. However, other studies also reported that MDD exhibited decreased fALFF values in the frontal lobe (49) and increased fALFF values in the temporal lobe relative to HCs (50). The discrepancy in the results may be explained by the differences in age, illness duration, treatment, data processing method, and population.

Additionally, in comparison to MDD, BD showed decreased fALFF values in the right cerebellar lobule VIII/IX and bilateral PCG, suggesting that different neural activities may exist between BD and MDD (6). In recent years, differentiating bipolar depression from MDD has been of great interest, but the biomarkers (electrophysiology, neuroimaging, or heredity) have not been established (51). In structural and functional brain studies, the alteration of cerebellum has been widely reported in BD and MDD (52). As a central component of the Default Mode Network (DMN), the posterior cingulate cortex (PCC) strongly connects with intrinsic connectivity networks such as the attention network and the fronto-parietal control network (53). One study reported that BD showed stronger FC in the left precuneus–left cerebellar lobule IX circuit and weaker FC in the right dorsolateral prefrontal cortex–left cerebellar lobule Crus I circuit than MDD (54). Han et al. reviewed that BD showed stronger FC in the DMN and reduced integrity of white matter tracts in the anterior part of the corpus callosum and posterior cingulum relative to MDD (6). As described above, the cerebellum and PCC are involved in emotion and cognition, and it seems that different brain activities in BD and MDD may result in different emotional and cognitive performances through different connectivity between brain regions. Moreover, SVM analysis showed that the fALFF value in the right cerebellar lobule VIII/IX may be a potential marker to identify BD patients at depressive state from MDD patients, with sensitivity, specificity and accuracy all more than 0.70.

We finally found that increased fALFF values in the right ITG of BD were negatively correlated with years of education (*r* = −0.490, *p* = 0.028), and decreased fALFF values in the right cerebellar lobule IV/V of BD were positively correlated with visuospatial abilities (*r* = 0.449, *p* = 0.047). Studies found that education plays a protective role in cortical volume (55) and is positively correlated with brain metabolism and FC in healthy elders (56). In comparison to HCs, better education in BD may serve as an important proxy of reserve to tolerate pathological effects through altering neural activity in the temporal lobe. As mentioned above, the cerebellum is regarded to play a role in the pathogenesis of depression (57) and in cognition. We consider that decreased neural activity observed in the cerebellum of BD may result in cognitive impairments including deficits in visuo-related executive function.

In this study, we observed different brain activity among HCs, BD patients, and MDD patients, and that altered brain activity in BD patients was correlated with cognition. These results preliminarily suggest that abnormal brain activity mediates BD and MDD and further influences cognition. Future studies can explore neural mechanisms in BD by performing different cognitive tasks and clarify how the brain regions functionally participate in emotion. Furthermore, exploring new drugs to correct the altered patterns of brain activity may be potentially helpful to treat BD and MDD patients in the future. The fALFF method is a sensitive approach to detect brain activity. However, the reliability of this approach for differential diagnosis in psychiatric diseases needs to be validated in the future.

The present study has some strengths and limitations. Firstly, all the patients with BD or MDD were first-episode, drug-naïve, and short-illness-duration patients, which may be helpful to explore the authentic etiologies and pathologies of BD and MDD for excluding the effects of treatment, disease subtype, and long disease course. Secondly, all the BD patients recruited were at a depressive episode, and the influence of disease state on the results can be partly controlled. Nonetheless, this study is limited by its small sample size in the patient groups. In addition, the fALFF approach we adopted in this study could not eliminate the influence of physiological noises (such as heartbeats and respiratory rhythms) by using a relatively low sampling rate (TR = 2 s), and we did not evaluate the gray/white matter anomalies before analyses. Besides, a previous study has reported that the eye status (eyes-open or eyes-closed) can influence the results of rs-MRI (58), but we did not control it in this study. Overall, this cross-sectional study is difficult to conclude whether abnormal fALFF values in BD and MDD patients are manifestations or causes of the diseases. Follow-up observations are required to verify the causalities.

## Data Availability Statement

The original contributions presented in the study are included in the article/supplementary material, further inquiries can be directed to the corresponding authors.

## Ethics Statement

The studies involving human participants were reviewed and approved by the Ethics Committee of the Second Xiangya Hospital of Central South University. The patients/participants provided their written informed consent to participate in this study.

## Author Contributions

HW designed the entire study protocol. JC, HT, JH, and HX recruited the participants. SL, ZT, KJ, CW, and XX collected the data. WG and BW analyzed the data and prepared all the figures and tables. YQ performed the MRI examination. MY and YQ were the major contributors in writing the manuscript. All authors read and approved the final manuscript.

## Funding

This study was supported by grants from the National Natural Science Foundation of China (Grant Nos. 81971258, 81270019, and 81501163), the Fundamental Research Funds for the Central Universities of Central South University (2021zzts390), and the Key-Area Research and Development Program of Guangdong Province (2018B030334001).

## Conflict of Interest

The authors declare that the research was conducted in the absence of any commercial or financial relationships that could be construed as a potential conflict of interest.

## Publisher's Note

All claims expressed in this article are solely those of the authors and do not necessarily represent those of their affiliated organizations, or those of the publisher, the editors and the reviewers. Any product that may be evaluated in this article, or claim that may be made by its manufacturer, is not guaranteed or endorsed by the publisher.

## Footnotes

^1^Available from: https://www.csie.ntu.edu.tw/~cjlin/libsvm/.
